# miR-138-5p Inhibits the Growth and Invasion of Glioma Cells by Regulating WEE1

**DOI:** 10.1155/2022/7809882

**Published:** 2022-01-28

**Authors:** Jianwu Gong, Zhi Tang, Zhengtao Yu, Zhiyong Deng, Yan Liu, Nianjun Ren, Lei Wang, Zhengwen He

**Affiliations:** ^1^Department of Neurosurgery, Hunan Cancer Hospital and the Affiliated Cancer Hospital of Xiangya School of Medicine, Central South University, No. 283 Tongzipo Road, Yuelu District, Changsha, 410006 Hunan, China; ^2^The Third Xiangya Hospital, Central South University, No. 138 Tongzipo Road, Yuelu District, Changsha, 410006 Hunan, China; ^3^Department of Neurosurgery, Haikou People's Hospital, The Affiliated Haikou Hospital of Xiangya School of Central South University, No. 43 Renmin Road, Meilan District, Haikou, 570208 Hainan, China; ^4^Department of Neurology, Changsha Central Hospital, University of South China, No. 161 Shaoshan Road, Yuhua District, Changsha, 410007 Hunan, China

## Abstract

**Background:**

Accumulating evidence has demonstrated the role of differentially expressed miRNAs in glioma progression. Our previous bioinformatics analyses revealed a role of miR-138-5p in glioma. miR-138-5p was decreased in various tumors, and He et al. found that miR-138-5p had an inhibitory effect on glioma cells in 2021. However, the role of miR-138-5p in the development of glioma and the underlying mechanism is unknown. In this study, we explored whether miR-138-5p affects the biology of glioma by regulating WEE1 expression.

**Methods:**

miR-138-5p and WEE1 G2 checkpoint kinase (WEE1) RNA and protein expression levels in glioma tissues were detected with qRT-PCR and western blotting, respectively. The effects of miR-138-5p and WEE1 on glioma cell migration and invasion were investigated using Transwell assays. CCK-8 assay was used to measure the effects of miR-138-5p and WEE1 on glioma cell proliferation. The mortality of glioma cells transfected with miR-138-5p and WEE1 was measured with flow cytometry. The relationship between miR-138-5p and WEE1 was explored using a luciferase reporter analysis.

**Results:**

Functional studies indicated that overexpression of miR-138-5p suppressed cell proliferation, migration, and invasion and promoted death in glioma cell lines. WEE1 was identified as a target of miR-138-5p, and overexpression of miR-138-5p significantly suppressed the levels of WEE1. Moreover, reintroduction of WEE1 partially abrogated miR-138-5p-induced suppression of motility and invasion in glioma cells.

**Conclusion:**

The low expression of miR-138-5p in glioma suggests a tumor suppressor role for this miRNA. miR-138-5p suppresses glioma progression by regulating WEE1. These data provide new insights into the molecular mechanism of glioma.

## 1. Introduction

Glioma is the most common primary intracranial tumor type, accounting for 81% of intracranial malignant tumors [1, 2]. Glioma remains a difficult cancer to treat despite the availability of surgical procedures, radiotherapy, temozolomide chemotherapy, and targeted therapies such as bevacizumab [3, 4]. Understanding the relevant molecular signaling pathways and identifying new biomarkers are important for improving the prognosis of glioma patients. MicroRNAs (miRNAs) are a class of small noncoding RNA molecules approximately 19–24 nucleotides in length, regulating mRNA expression by binding to the 3′-untranslated region (3′-UTR) [5, 6], altering cell proliferation, cell cycle, apoptosis, and differentiation. In tumors, miRNAs are ubiquitous and can play both a procancer and anticancer role by regulating the expression of specific signaling molecules [7].

Many miRNAs have been identified in glioma through the wide application of second-generation sequencing [8, 9]. In previous studies, we identified nine glioma-associated miRNAs: miR-338-3p, miR-137, miR-128-3p, miR-218-5p, miR-7-5p, miR-139-5p, miR-124-3p, miR-383-5p, and miR-138-5p [10]. We found that high expression of miR-137 and miR-338-3p was associated with decreased overall survival (*P* < 0.01). Low expression of miR-139-5p and miR-138-5p was associated with poor overall survival (*P* < 0.01) [10]. We showed that miR-139-5p exerted antitumor effects in glioma by directly targeting GABRA1 [10]. Therefore, we hypothesize that miR-138-5p plays an important role in the malignant biology of glioma and can serve as a diagnostic and novel target for the treatment of glioma.

The biological roles and potential mechanisms of miRNA in gliomas are largely unknown, requiring further research. In this study, we investigated the expression and function of miR-138-5p. We found that miR-138-5p may play a tumor-suppressive role by targeting WEE1, which acts as an oncogene in glioma.

## 2. Materials and Methods

### 2.1. Cell Lines

The human glioma cell lines U87 and U251 from BeNa Culture Collection (BNCC, China) were cultured in vitro in Dulbecco's Modified Eagle's Medium (DMEM, Sigma, USA) containing 10% fetal bovine serum (FBS, Gibco, USA) and antibiotics (100 U/mL streptomycin and 100 U/mL penicillin). Cells were cultured at 37°C in an incubator containing 5% CO_2_.

### 2.2. Cell Transfection

Negative control (NC), miR-138-5p mimic, and pcDNA3.1-WEE1 plasmids overexpressing WEE1 were purchased from Honorgene Co., Ltd. (Changsha, Hunan, China). Cells were transfected using Lipofectamine 2000 reagent (Invitrogen, Carlsbad, CA, USA) according to the manufacturer's protocol.

### 2.3. Quantitative Real-Time Polymerase Chain Reaction (qRT-PCR)

Total RNA was extracted from cultured U87 or U251 cells 6 h after transfection using Trizol total RNA extraction reagent (Thermo, USA) according to the manufacturer's recommendation. The reverse transcription 200 ng total RNA was performed using a SuperRT RT reagent kit (CWBio, China) and a SYBR PCR Master Mix (CWBio, China) quantitative real-time PCR. The PCR was set as initial denaturation at 95°C for 10 min, 95°C for 15 s, and 60°C for 30 s, with a total of 40 cycles. All experiments were carried out in triplicate. Expression fold changes were calculated using the 2−*^ΔΔ^*Ct method. All primer sequences are listed in Table 1.

### 2.4. Western Blot Analysis

U87 or U251 cells 6 h after transfection were lysed by lysis buffer with RIPA on ice for 30 min and centrifuge at 4°C for 20 min at 12000*g*/min. The total protein concentration of cell lysates was assessed using the BCA Protein Assay kit. Subsequently, 20 *μ*g protein samples were separated on a 10% SDS-PAGE by the use of a Bio-Rad Bis-Tris Gel system (Bio-Rad, CA, USA), transferred onto polyvinylidene difluoride membranes (Millipore, Danvers, MA, USA), blocked with 5% nonfat milk for 60 min at room temperature. Primary antibodies were WEE1 (1 : 1000, Proteintech, USA) and *β*-actin (1 : 5000, Proteintech, USA); corresponding secondary antibodies were anti-mouse and anti-rabbit (Proteintech, USA). Primary antibodies against WEE1 were purchased from Bioss Co., Ltd. (Beijing, China) and the primary antibodies against N-cadherin, vimentin, and *β*-actin (Proteintech, USA). Finally, the proteins were detected by enhanced chemiluminescence (ECL) and then exposed about 60 s for scanning and measuring.

### 2.5. Gene Analysis

OncoLnc is an independent software tool used to analyze the expression of genes and proteins in tumors (http://www.oncolnc.org/). FunRich was used to predict downstream target genes of mir-138-5p (http://www.funrich.org/) [11].

### 2.6. Cell Viability Assays

Using Cell Counting Kit-8 (CCK-8; Tongren, Japan) to test cell proliferation, U87 or U251 cells 6 h after transfection were seeded on 96-well plates and cultured with 100 *μ*L cell culture medium. After culturing for 48 h, 10 *μ*L CCK-8 was added into cell culture medium and incubated at 37°C for 4 h. Calculate the absorbance at 450 nm with a plate reader (BioTek; Huisong, Shenzhen, China).

### 2.7. Colony Formation Assays

U87 or U251 cells 6 h after transfection were inoculated in 6-well plates (Nunc, Roskilde, Denmark) at a density of 400 cells per well. After 14 d culturing, the cell colonies were fixed with ethanol, treated with 0.3% crystal violet solution (Sangon, Songjiang, Shanghai, China) for 0.5 h, and washed twice with deionized water.

### 2.8. Cell Apoptosis Assay

Mortality of U87 or U251 cells 6 h after transfection was detected by apoptosis detection kit (KeyGen BioTECH, China). The cells were washed with PBS (Hyclone, USA) for three times and centrifuged. The collected cells were suspended with 500 *μ*L binding buffer. Then, 5 *μ*L Annexin V-APC was added for mixing, and 5 *μ*L propidium iodide was added for mixing. The reaction was carried out at room temperature under dark conditions for 10 min and detected by flow cytometry within 1 h.

### 2.9. Transwell Assays

Transwell assay was used to assess migration and invasion of U87 or U251 cells 6 h after transfection. Transwell chamber (Corning, Shanghai, China) was used to measure migration. The Matrigel (BD, USA) melted at 4°C overnight. 100 *μ*L of diluted Matrigel was then added in the chamber. Afterwards, 200 *μ*L of serum-free medium was added to the upper chamber; meanwhile, 500 *μ*L of 10% FBS DMEM was added to the lower chambers, and 2 × 10^5^ collected cells in total were planted in the upper ones and cultivated in the incubator for another 48 h. Subsequently, the invading chamber was taken out, and cells on the polycarbonate membrane were fixed with 4% paraformaldehyde, followed by staining with 0.1% crystal violet. The absorbance (OD) was measured at 550 nm by microplate analyzer in three random fields after acetic acid immersion and decolorization. The results were repeated three times.

### 2.10. Dual Luciferase Reporter Assays

The Luciferase Detection System Kit (Promega, Madison, WI, USA) was used to detect luciferase activity in U87 and U251 cells in logarithmic growth phase. Human WEE1 gene wild-type 3′-untranslated region (UTR) or mutant 3′-UTR sequence of WEE1 (WEE1 WT or WEE1 MUT) double luciferase expression plasmid was purchased from Honorgene (Aono Genomics, Changsha Abiwei Biotechnology Co., Ltd.). The cells were inoculated in a 24-well plate and cotransfected with Lipofectamine 2000 reagent (Invitrogen Co., Carlsbad, CA, USA) and WEE1 expression vector with NC simulators or miR-138-5p simulators. The cells were inoculated on a 24-well plate. Lipofectamine 2000 reagent (Invitrogen, Carlsbad, CA, USA) and WEE1 expression vector cotransfect NC simulators or miR-138-5p simulators. After incubation for 48 hours according to the manufacturer's instructions, the transfected cells were collected and used for luciferase activity determination according to the protocol in the Dual-Glo Luciferase Assay System kit.

### 2.11. Statistical Analysis

All data are presented as mean ± SD of three independent experiments. Quantitative data were compared with *t*-test. *P* < 0.05 was considered statistically significant. Statistical and graphical analyses were performed using GraphPad Prism 5. Figures were created using Adobe Illustrator software.

## 3. Results

### 3.1. Overexpression of miR-138-5p Inhibits Glioma Cell Proliferation and Induces Cell Death

**Figure 1:**
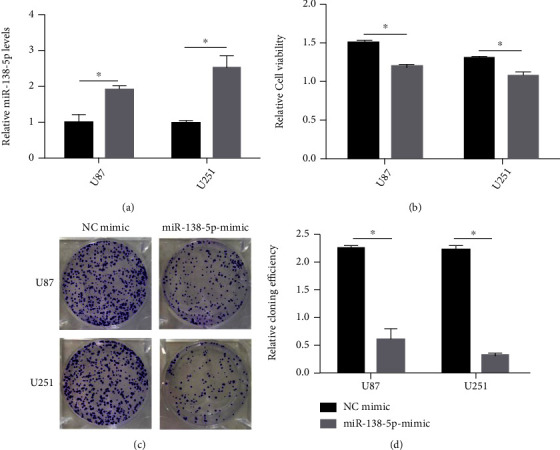
Effects of miR-138-5p on proliferation and apoptosis of U87 and U251 cells. (a) miR-138-5p transfection increased miR-138-5p levels in U87 and U251 cells compared to NC-transfected cells. (b) Cell viability of U87 and U251 cells was determined by a CCK-8 assay. (c, d) Colony formation experiments showed that the absorbance (OD value) of U87 and U251 cells decreased at 550 nm after forced expression of miR-138-5p. Each experiment was repeated at least three times. ^∗^*P* < 0.05.

**Figure 2:**
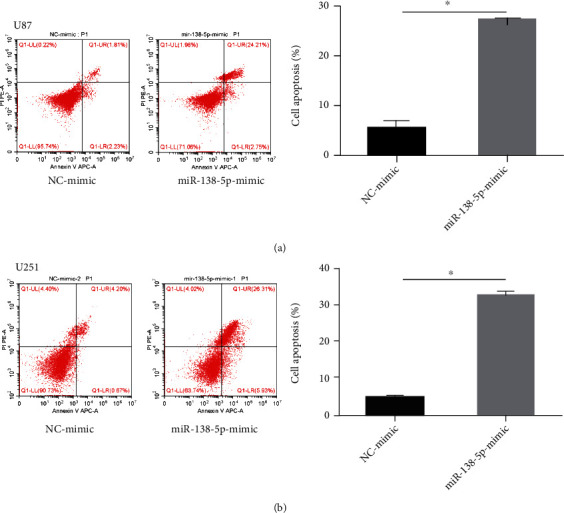
Effects of miR-138-5p on proliferation of U87 and U251 cells. (a) Flow cytometry was used to evaluate apoptosis rates in U87 cells transfected with NC or miR-138-5p mimics. (b) Flow cytometry was used to evaluate apoptosis rates in U251 cells transfected with NC or miR-138-5p mimics. Each experiment was repeated at least three times. ^∗^*P* < 0.05.

To study the function of miR-138-5p in glioma, U87 and U251 cells were transfected with a miR-138-5p mimic. The transfection efficiency was confirmed using qRT-PCR. After transfection with a miR-138-5p mimic, the relative expression of miR-138-5p in U87 and U251 cells was approximately doubled (Figure 1(a)). A CCK-8 assay showed that overexpression of miR-138-5p significantly reduced the cell viability of U87 and U251 cells (Figure 1(b)). In addition, colony formation experiments showed that the absorbance of U87 and U251 cells decreased at 550 nm after forced expression of miR-138-5p (Figures 1(c) and 1(d)). Flow cytometry confirmed that mortality in U87 and U251 cells was significantly increased with miR-138-5p mimic transfection (Figure 2). miR-138-5p promotes cell death of U87 and U251. Taken together, these results suggest that miR-138-5p plays an inhibitory role in the development of glioma.

### 3.2. Ectopic Expression of miR-138-5p Impedes the Migration and Invasion of Glioma Cells

**Figure 3:**
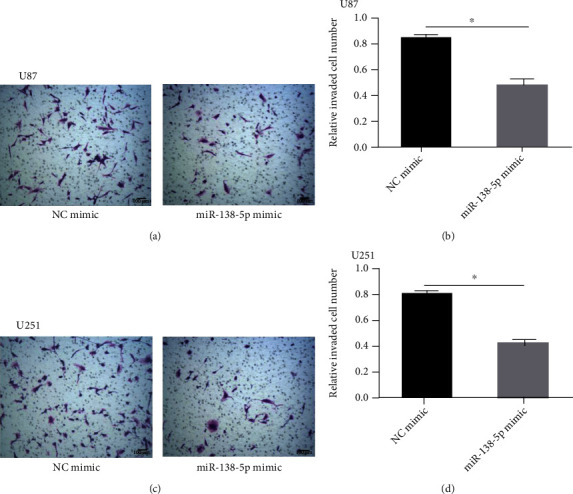
Overexpression of miR-138-5p inhibited the migration and invasion of U87 and U251 cells. (a–d) Transwell migration and invasion assays with U87 and U251 cells transfected with miR-138-5p mimics or NC. Each experiment was repeated at least three times. ^∗^*P* < 0.05.

**Figure 4:**
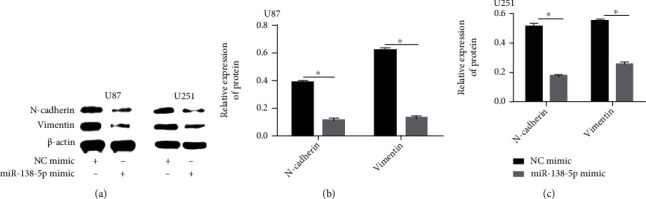
Protein levels of vimentin and N-cadherin. (a–c) Western blotting was used to detect protein levels of vimentin and N-cadherin 6 h after transfection. Each experiment was repeated at least three times. ^∗^*P* < 0.05.

A Transwell assay was utilized to evaluate the effect of miR-138-5p on migration and invasion of U251 and U87 cells. Overexpression of miR-138-5p significantly reduced the invasion ability of U251 and U87 cells (Figure 3). Protein levels of epithelial-to-mesenchymal-related molecules were measured using western blotting. N-cadherin and vimentin were markedly reduced after transfection with a miR-138-5p mimic in U87 and U251 cells (Figures 4(a)–4(c)). Our data suggest that enhanced miR-138-5p expression impairs the metastatic potential of glioma cells.

### 3.3. miR-138-5p Regulates WEE1 Expression

**Figure 5:**
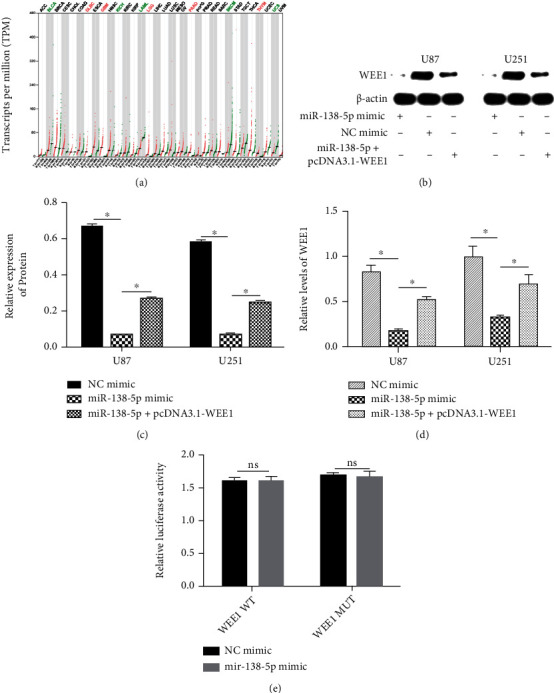
WEE1 is targeted by miR-138-5p at the 3′-UTR. (a) Prediction of WEE1 expression in tumors by OncoLnc. (b, c) Western blotting showed that transfection with a miR-138-5p mimic reduced the levels of WEE1 protein in U87 and U251 cells. (d) qRT-PCR showed that the miR-138-5p mimic transfection reduced the levels of WEE1 mRNA in U87 and U251 cells. (e) Luciferase activity in U87 and U251 cells was measured using dual luciferase reporter genes. Each experiment was repeated at least three times. ^∗^*P* < 0.05.

We next wanted to determine the molecular mechanism underlying the tumor-suppressive effects of miR-138-5p in glioma. A previous bioinformatics analysis suggested that miR-138-5p may play a role in the growth of glioma. The target genes of miR-138-5p in human cells were predicted by an online bioinformatics database (FunRich, http://www.funrich.org/). Among these candidate genes, WEE1 was selected as a potential target gene because it has been shown to be involved in the regulation of multiple functions in various cancer types (Figure 5(a)). The protein levels of WEE1 as measured by western blotting were significantly reduced in U251 and U87 cells transfected with a miR-138-5p mimic. Overexpression of miR-138-5p significantly reduced WEE1 expression, as measured by qRT-PCR (Figure 5(d)). To verify whether WEE1 is the target of miR-138-5p, a dual luciferase reporter gene assay was performed in U87 and U251 cells. The relative luciferase activity of the WEE1 3′-UTR (WEE1-WT) or WEE1 3′-UTR (WEE1-MUT) mutants and the miR-138-5p mimic showed no significant changes with cotransfection compared with the NC (Figure 5(e)). Contrary to what the database predicted, the luciferase experiment did not confirm WEE1 as a direct regulatory target of miR-138-5p, and there may be an indirect regulatory mechanism between them.

Overexpression of WEE1 reverses the inhibitory effect of miR-138-5p on the proliferation and invasion of glioma cells and induced death.

We investigated whether WEE1 can mediate the effects of miR-138-5p on proliferation, invasion, and death in glioma cells. The rescue experiment was performed by cotransfection of a miR-138-5p mimic with or without a pcDNA3.1-WEE1 plasmid. qRT-PCR analysis confirmed the reduction of WEE1 mRNA levels with the overexpression of miR-138-5p (Figure 5(d)). Transfection of the pcDNA3.1-WEE1 plasmid subdued the inhibition of miR-138-5p on WEE1 expression (Figure 5(d)). Western blotting demonstrated that miR-138-5p overexpression reduced the protein levels of WEE1 (Figures 5(b) and 5(c)).

**Figure 6:**
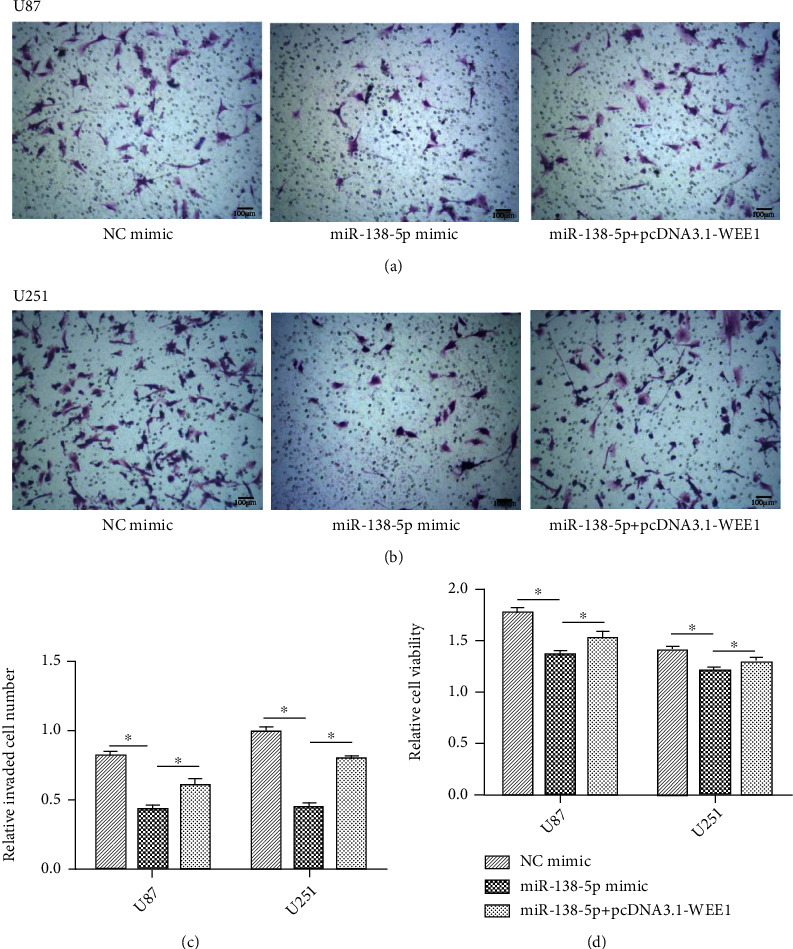
WEE1 expression inhibited the inhibitory effect of miR-138-5p on glioma cells. (a–c) A Transwell invasion assay was used to assess the number of invaded U87 and U251 cells under various experimental conditions. (d) A CCK-8 assay was used to assess the proliferation of U87 and U251 cells in different groups. Each experiment was repeated at least three times. ^∗^*P* < 0.05.

The invasion assay showed that pcDNA3.1-WEE1 transfection significantly reversed the inhibitory effect of miR-138-5p on the invasion ability of U87 and U251 cells and restored the expression of WEE1 (Figures 6(a)–6(c)).

**Figure 7:**
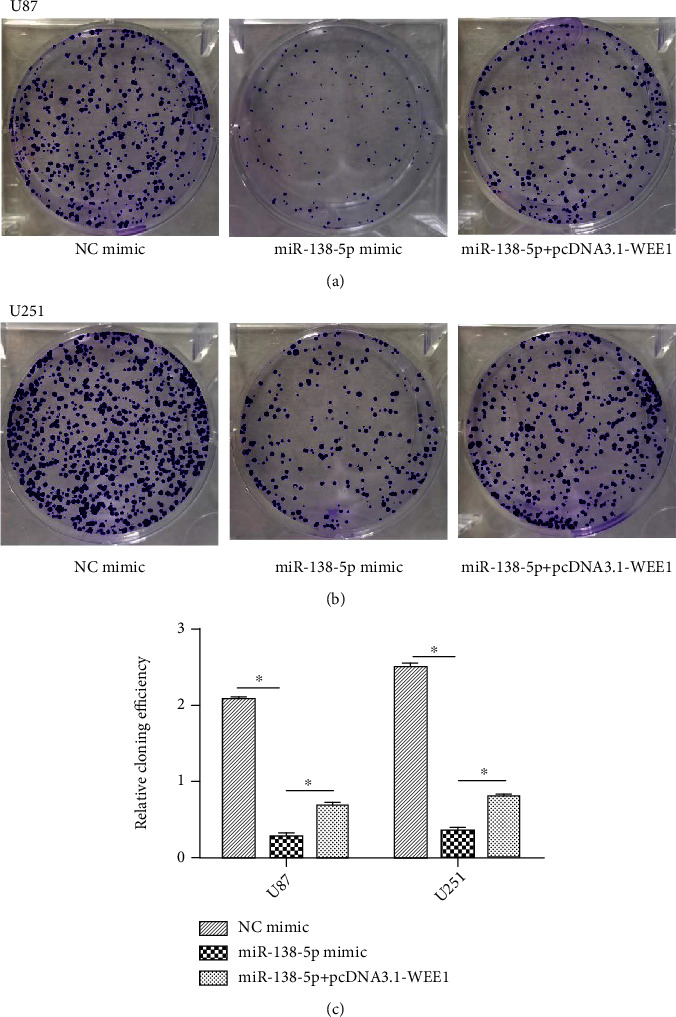
The expression of WEE1 inhibited miR-138-5p on proliferation of glioma cells. (a, b) Comparison of U87 and U251 cell colonies two weeks after transfection of NC mimic, miR-138-5p mimic, and miR-138-5P+pcDNA3.1-WEE1. (c) The reintroduction of WEE1 significantly increased the number of colonies in U87 and U251 cells compared with the miR-138-5P mimic alone transfection group. Each experiment was repeated at least three times. ^∗^*P* < 0.05.

We performed a CCK-8 assay to measure proliferation in our system. Ectopic expression of miR-138-5p resulted in a significant reduction in viability (Figure 6(d)). Conversely, cotransfection of the miR-138-5p mimic and pcDNA3.1-WEE1 reversed the inhibition of miR-138-5P mimic on U87 and U251 cells (Figure 6(d)). In addition, the reintroduction of WEE1 significantly increased the number of colonies in U87 and U251 cells compared with the miR-138-5P mimic alone transfection group (Figure 7).

**Figure 8:**
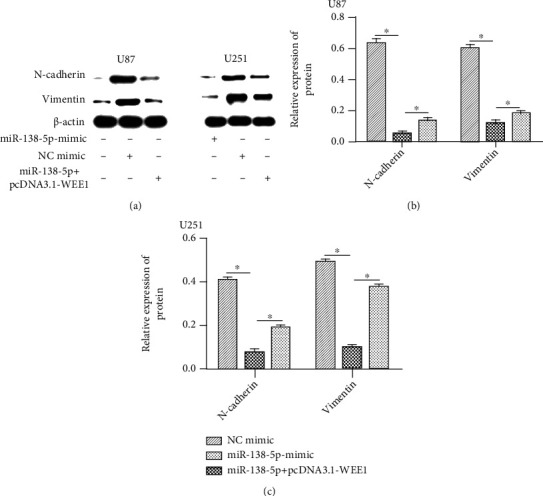
The expression of WEE1 inhibited miR-138-5p-induced N-cadherin and vimentin expression. (a) Expression of N-cadherin and vimentin in U87 and U251 cells in each treatment group was determined using western blot. (b, c) The inhibition of N-cadherin and vimentin expression was reversed with the cotransfection of miR-138-5p and pcDNA3.1-WEE1. Each experiment was repeated at least three times. ^∗^*P* < 0.05.

We measured the protein (N-cadherin and vimentin) levels of epithelial-to-mesenchymal-related molecules using western blotting. N-cadherin and vimentin were significantly reduced after transfection with a miR-138-5p mimic in U87 and U251 cells (Figures 5(b) and 5(c)). The inhibition of N-cadherin and vimentin expression was reversed with the cotransfection of miR-138-5p and pcDNA3.1-WEE1 (Figure 8).

**Figure 9:**
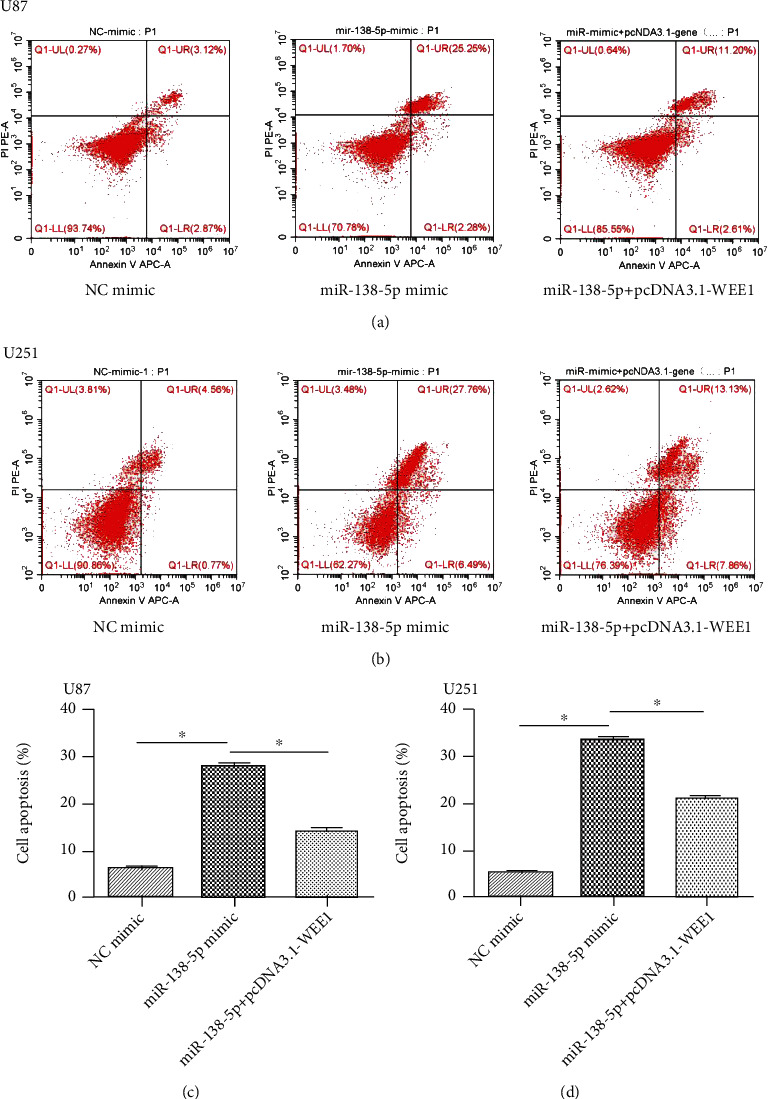
WEE1 expression reduced miR-138-5p-induced apoptosis in U87 and U251 cells. (a, b) The mortality of U87 and U251 cells in each group was determined by flow cytometry. (c, d) Compared with NC mimic, miR-138-5p mimic promoted apoptosis of U87 and U251 cells. And in U87 and U251 cells, the apoptosis rate of transfected miR-mimic+pcDNA3.1-WEE1 was lower than that of transfected miR-138-5p mimic alone. Each experiment was repeated at least three times. ^∗^*P* < 0.05.

Flow cytometry confirmed that the mortality of U87 or U251 cells was significantly increased after miR-138-5p overexpression (Figure 2). The enhancing effect of miR-138-5p on cell death was weakened after pcDNA3.1-WEE1 plasmid transfection (Figure 9). This suggests that pcDNA3.1-WEE1 attenuates the inhibitory effect of miR-138-5p on glioma cells.

In conclusion, our study demonstrated that WEE1 is a downstream functional effector of miR-138-5p in glioma cells, and miR-138-5p regulates the development and progression of glioma through WEE1.

## 4. Discussion

Glioma is the most common intracranial malignant tumor and has a very low survival rate [1, 2]. To better understand the pathogenesis of glioma and to discover more effective treatments, it is necessary to identify valuable targets [12]. Various molecular biomarkers of glioma have been identified in recent decades [13]; however, they are rarely used in clinical practice for relatively low sensitivity [14, 15]. Recently, miRNAs have attracted the attention of scientists as a new clinical biomarker [16].

Our previous research showed that low expression of miR-139-5p and miR-138-5 was associated with poor overall survival (*P* < 0.01) [10]. In this study, we focused on the association between miR-138-5p and WEE1 in order to explore the potential mechanism of the antitumor effects of miR-138-5p in glioma. We confirmed that the ectopic expression of WEE1 reversed the inhibitory effect of miR-138-5p overexpression on proliferation, migration, and invasion of glioma cells. However, a miR-138-5p mimic transfection did not significantly reduce the luciferase activity and WEE1 protein expression in glioma cells. This lack of a reduction of luciferase activity and WEE1 protein expression could be because of the indirect regulatory relationship between the two molecules. These data suggest that miR-138-5p plays a tumor-suppressive role by regulating WEE1.

MicroRNAs do not express proteins. However, it plays a role by regulating the expression of various genes [17]. miR-138-5p, a functional miRNA located on chromosome 3p21.32, is involved in different tumor types [18–22], showing abnormally low in a variety of malignant tumors, such as osteosarcoma [18], gallbladder carcinoma [19], undifferentiated thyroid carcinoma [20], non-small-cell lung cancer [21], and oral squamous cell carcinoma [22].

Recent studies have shown that the upregulation of miR-138-5p in tumor cells may lead to the reversal of the malignant phenotype [23]. There is lower expression of miR-138-5p in glioma tissue than in normal brain tissue [24]. miR-138-5p can inhibit angiogenesis in glioma [25]. LINC00174 can promote the development of glioma by downregulating the expression of mir-138-5p [26]. SNHG7 may enhance the proliferation of glioma by regulating the miR-138-5p/EZH2 signal axis [27]. However, the role of miR-138-5p in the clinical diagnosis and treatment of glioma is still limited, and therefore, it is necessary to further explore its related targets. Our data suggest that miR-138-5p mainly acts as a tumor suppressor in glioma.

WEE1, a member of the tyrosine kinase family, is a G2/M checkpoint regulatory protein involved in the regulation of cell cycle [28]. Recently, many studies have shown that WEE1 is upregulated in numerous tumors and plays a cancer-promoting role [29, 30]. This indicates that WEE1 may be a good candidate target for tumor therapy. There are some studies that have shown that WEE1 is overexpressed in gliomas. WEE1 promotes the proliferation, migration, and invasion of glioma cells [31, 32]. WEE1 is overexpressed in pediatric high-grade gliomas, with increasing expression positively correlated with malignancy [33]. Wu et al. showed that in GBM, WEE1 inhibition abrogated the G2/M arrest and propelled cells to prematurely enter into mitosis and consequent cell death through mitotic catastrophe and apoptosis [34]. The underlying mechanism in glioma progression remains unclear.

Future studies include fully describing the mechanism of miR-138-5p and WEE1 in glioma and exploring more candidate therapeutic targets of miR-138-5p.

## 5. Conclusion

miR-138-5p regulates proliferation, migration, and invasion of glioma cells by targeting WEE1, which is associated with poor prognosis of glioma patients. miR-138-5p may be a potential therapeutic target for glioma patients.

## Figures and Tables

**Table 1:** Primers for qRT-PCR.

Gene	Sequences (5′-3′)
miR-138-5p (F)	CTCGCTTCGGCAGCACA
miR-138-5p (R)	AACGCTTCACGAATTTGCGT
WEE1 (F)	GCTTCCCTCACAGTGGTATG
WEE1 (R)	CCGAGGTAATCTACCCTGTCTGA
GAPDH (F)	GGTCTCCTCTGACTTCAACA
GAPDH (R)	GCCAAATTCGTTGTCATAC

Abbreviation: miR-138-5p: microRNA-138-5p; qRT-PCR: quantitative real-time polymerase chain reaction; GAPDH: glyceraldehyde 3-phosphate dehydrogenase.

## Data Availability

All the data is available.
